# *Ex vivo*-generated human CD1c^+^ regulatory B cells by a chemically defined system suppress immune responses and alleviate graft-versus-host disease

**DOI:** 10.1016/j.ymthe.2024.10.026

**Published:** 2024-10-26

**Authors:** Yingying Bao, Jialing Liu, Zhishan Li, Yueming Sun, Junhua Chen, Yuanchen Ma, Gang Li, Tao Wang, Huanyi Liu, Xiaoran Zhang, Rong Yan, Zhenxia Yao, Xiaolu Guo, Rui Fang, Jianqi Feng, Wenjie Xia, Andy Peng Xiang, Xiaoyong Chen

**Affiliations:** 1 Center for Stem Cell Biology and Tissue Engineering, Key Laboratory for Stem Cells and Tissue Engineering, Ministry of Education, Sun Yat-sen University, Guangzhou 5100080, China; 2 National-Local Joint Engineering Research Center for Stem Cells and Regenerative Medicine, Zhongshan School of Medicine, Sun Yat-sen University, Guangzhou 5100080, China; 3 Institute of Gene and Cell Therapy, Xi’an Jiaotong University, Xi’an 710061, China; 4 Department of Gastrointestinal Surgery, The First Affiliated Hospital, Sun Yat-sen University, Guangzhou 510080, China; 5 Guangdong Provincial Key Laboratory of Liver Disease Research, The Third Affiliated Hospital, Sun Yat-sen University, Guangzhou 510630, China; 6 Center for Stem Cells Translational Medicine, Shenzhen Qianhai Shekou Free Trade Zone Hospital, Shenzhen 518067, Guangdong, China; 7 Institute of Blood Transfusion, Guangzhou Blood Centre, Guangzhou 510095, China

**Keywords:** regulatory B cells, graft-versus-host disease, cAMP-response element-binding protein, IL-10, mesenchymal stromal cells, protein kinase A, CD1c

## Abstract

IL-10^+^ regulatory B cells (Bregs) show great promise in treating graft-versus-host disease (GVHD), a life-threatening complication of post-hematopoietic stem cell transplantation. However, obtaining high-quality human IL-10^+^ Bregs *in vitro* remains a challenge due to the lack of unique specific markers and the triggering of pro-inflammatory cytokine expression. Here, by uncovering the critical signaling pathways in Breg induction by mesenchymal stromal cells (MSCs), we first established an efficient Breg induction system based on MSCs and GSK-3β blockage (CHIR-99021), which had a robust capacity to induce IL-10^+^ Bregs while suppressing tumor necrosis factor α (TNF-α) expression. Furthermore, these Breg populations could be identified and enriched by CD1c^+^. Mechanistically, MSCs induced the expansion of Bregs through the PKA-mediated phosphorylation of cAMP response element-binding protein (CREB). Thus, we developed a chemically defined inducing protocol by PKA-CREB agonist, instead of MSCs, which can also effectively induce CD1c^+^ Bregs with lower TNF-α expression. Importantly, induced CD1c^+^ Bregs suppressed the proliferation of peripheral blood mononuclear cells and the inflammatory cytokine secretion of T cells. When adoptively transferred into a humanized mouse model of GVHD, induced CD1c^+^ Bregs effectively alleviated GVHD. Overall, we established an efficient *ex vivo* induction system for human Bregs, which has implications for developing novel Bregs-based therapies for GVHD.

## Graphical abstract

**Figure:**
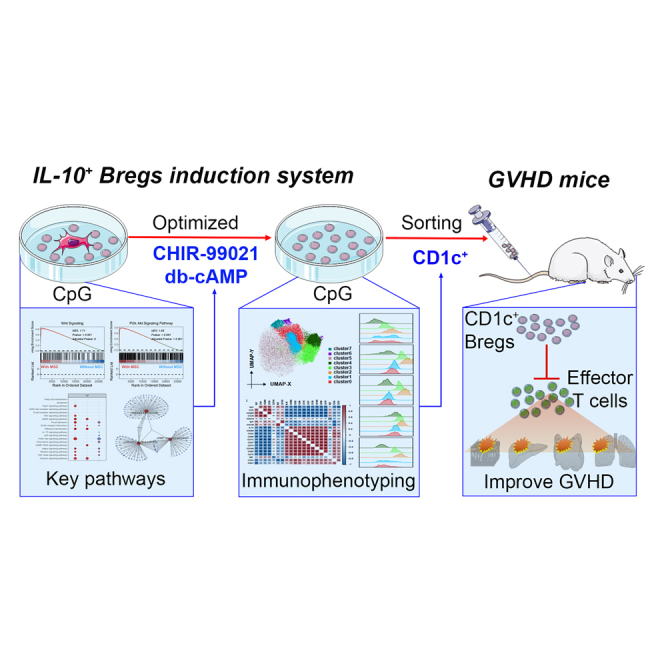

## Teaser

This study established a chemically defined induction system for IL-10^+^ Bregs and found that CD1c is a good marker for enriching the induced Bregs. Induced CD1c^+^ Bregs had immunosuppressive function and exhibited a powerful effect on alleviating GVHD, providing new ideas for the prophylaxis and treatment of GVHD.

## Introduction

Allogeneic hematopoietic stem cell transplantation (HSCT) is still the most effective therapeutic option for hematological malignancies in clinic, but graft-versus-host disease (GVHD) after HSCT limits the success of a potentially curative transplant. Around 50% of patients will develop GVHD, becoming the main cause of non-relapse death after HSCT.^1^ At present, the first-line treatment for GVHD is still glucocorticoids alone or in combination with immunosuppressants; however, 30%–50% of acute GVHD patients and 60%–80% of chronic GVHD patients do not respond to the treatment.^2^ Moreover, long-term use of these drugs usually causes some adverse effects, such as increased risk of infection, hyperglycemia, diarrhea, and osteoporosis.^3^ Several new approaches have been reported recently for steroid-refractory GVHD, including small-molecule inhibitors, antibodies, and cytokines^3^; however, their tolerability and efficacies need to be confirmed by plentiful trials. Therefore, novel and effective therapies for GVHD are urgently needed.

Recently, cell therapy has emerged as a potential and effective option for controlling GVHD. Regulatory B cells (Bregs) have become a potential candidate of cell therapy for GVHD, largely owing to their immunoregulatory capacity to downregulate the number and the function of effector T cells, monocytes, and so forth, and induce regulatory T cells.^4^ Bregs are closely related to GVHD. Patients with GVHD had a loss of Bregs compared with those without GVHD after HSCT. Patients who received HSCT with cord blood, containing many Bregs, developed a lower rate of GVHD^5^; and studies have shown that depletion of Bregs significantly augmented GVHD severity in mice.^6^^,^^7^ Our previous study and several other reports have demonstrated that mesenchymal stromal cells (MSCs) are likely to boost Bregs in improving GVHD and other diseases *in vivo*,^8^^,^^9^^,^^10^ such as systemic sclerosis and systemic lupus erythematosus. Collectively, Bregs play a critical role in preventing and treating GVHD, and the elevation of Bregs might be a potential approach for the prophylaxis and treatment of GVHD. Results from adoptive transfer studies further confirm the feasibility and effectiveness of Bregs-based therapy in GVHD,^11^^,^^12^^,^^13^^,^^14^ promising to be an ideal therapy for GVHD.

The acquisition of human Bregs *in vitro* is still challenging, however, with there being a limited number in peripheral blood and an incomplete understanding of their development.^15^ Bregs are inducible *in vitro* after stimulation, but they predominantly elicit pro-inflammatory signals in their activation and significantly increase proinflammatory cytokines such as tumor necrosis factor α (TNF-α), which in turn exert a pro-inflammatory effect.^16^^,^^17^ In addition, although multiple Breg phenotypes have been described, there is no suitable specific marker to enrich for induced interleukin-10^+^ (IL-10^+^) B cells. In recent years, MSCs have been reported to regulate the survival, proliferation, differentiation, and chemotaxis of B cells by cell-to-cell contact, soluble factors, and extracellular vesicles.^18^ Our previous results demonstrated that MSCs not only induced the IL-10^+^-producing Bregs but they also enhanced their survival and regulatory function *in vitro*,^8^^,^^19^ suggesting that MSCs-based Breg induction is a potential solution for the acquisition of human Bregs *in vitro*. However, the functional heterogeneity of mesenchymal stem cells causes their effects to be unstable or even contradictory,^20^^,^^21^ limiting their application.

In the present study, we established an efficient induction system for IL-10^+^ Bregs based on MSCs combined with glycogen synthase kinase-3β (GSK-3β) blockage and found that CD1c^+^ B cells could enrich the induced Bregs. Moreover, to overcome the functional heterogeneity of MSCs, we successfully revealed that protein kinase A (PKA)-mediated phosphorylation of cyclic AMP (cAMP) response element-binding protein (CREB) was the pivotal modulator for inducing Bregs in MSC-based systems. Thus, we optimized the Bregs induction system by enhancing PKA/CREB signaling in the absence of MSCs and found that this chemically defined protocol showed a similar efficiency in inducing CD1c^+^ Bregs with lower TNF-α expression. Induced CD1c^+^ Bregs had immunosuppressive function and exhibited a powerful effect on alleviating GVHD, providing novel cell-based therapy for GVHD.

## Results

### IL-10-producing Bregs were efficiently induced by MSCs combined with GSK-3β inhibitor

**Figure 1:**
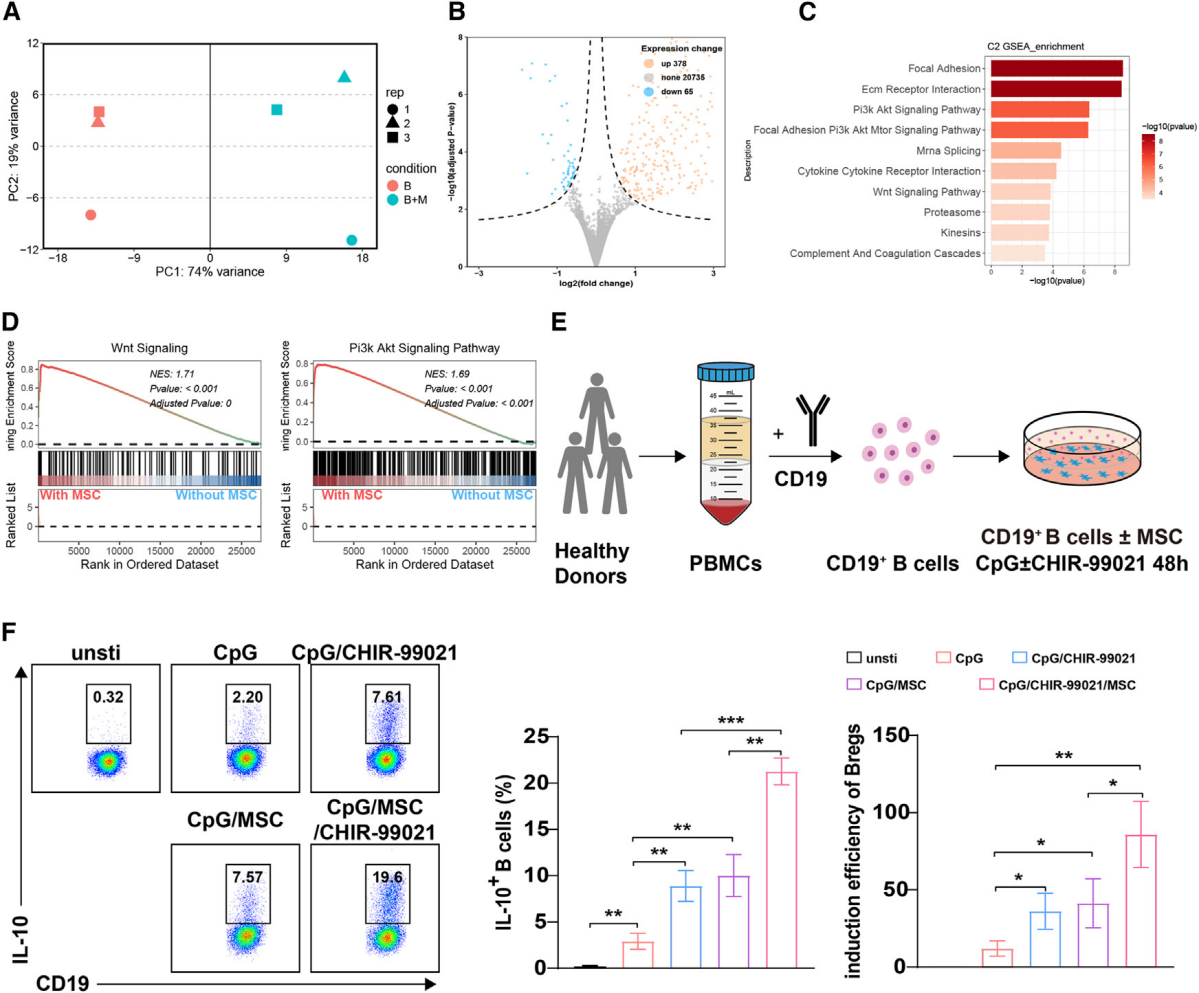
MSCs combined with CHIR-99021 had a robust capacity to induce IL-10^+^ B cells (A) Principal-component analysis of gene expression profiles across all B cell samples. (B) Volcano plots illustrating genes exhibiting significant differential expression (DESeq2 analysis) between B cells cultured alone and B cells cocultured with MSCs. (C) Gene set enrichment analysis (GSEA) of upregulated differentially expressed genes (DEGs). (D) Enrichment plots show the PI3K/Akt and Wnt signaling pathways in B cells alone or with MSCs. (E) The workflow of inducing IL-10^+^ B cells. (F) IL-10 production by unstimulated B cells and B cells stimulated by CpG, MSCs, and CHIR-99021 (a GSK-3β inhibitor, 10 μM) individually or in combination, was detected by flow cytometry. Quantification of IL-10-producing B cells and the induction efficiency of IL-10^+^ B cells in above treatment compared with unstimulated B cells. Data represent mean ± SEM of three independent experiments; not significant (ns) *p* ≥ 0.05; ∗*p* < 0.05; ∗∗*p* < 0.01; ∗∗∗*p* < 0.001; ∗∗∗∗*p* < 0.0001.

To establish an efficient MSC-based Breg induction system, we first analyzed the RNA sequencing (RNA-seq) data of MSC-induced IL-10^+^ producing Bregs in a B/MSCs coculture system, and we found that significant alterations in gene expression of B cells after induction with MSCs (Figures 1A and 1B). Using gene set enrichment analysis (GSEA) on the differentially expressed genes (DEGs), we found that some key signaling pathways were involved in this process, including extracellular matrix receptor interaction and Wnt and phosphatidylinositol 3-kinase (PI3K)/Akt signaling pathways (Figures 1C and 1D). Since the Wnt and PI3K/Akt signaling pathways have been reported to regulate IL-10 expression in immune cells,^22^^,^^23^ we prioritized verification of whether they are essential for the induction of IL-10^+^ Bregs. The results showed that only CHIR-99021, a GSK-3β inhibitor that functions as a Wnt activator, could induce the IL-10 expression in B cells, and the combination with MSCs significantly enhanced the inducing effects (Figure S1A). Moreover, when CHIR-99021 was added to the CpG-stimulated system (a conventional Bregs induction), the IL-10^+^ Bregs were markedly induced, and 10 μM was an appropriate concentration, with a higher IL-10^+^ Breg and survival rate compared with 5, 20, and 50 μM (Figure S1B).

Thus, we tried to establish human Breg-inducing systems based on Toll-like receptor 9 (TLR9) agonist (CpG), MSCs, and GSK-3β inhibitor (CHIR-99021), individually and in combination (Figure 1E). In these inducing systems, we found that either CHIR-99021 or MSCs could significantly increase the proportion of IL-10^+^ B cells in the conventional Breg-inducing system with CpG (Figure 1F). Interestingly, MSCs combined with CHIR-99021 could further increase IL-10^+^ B cells compared with MSCs alone, and it drove nearly a 100-fold generation of IL-10^+^B cells (Figure 1F). In addition, we found that the combination of MSCs and CHIR-99021 not only enhanced the IL-10 production but also decreased the proinflammatory cytokines such as TNF-α during induction (Figures S2A and S2B) and increased the percentage of TNF-α^−^IL-10^+^ B cells (Figure S2B). Furthermore, this induction system also promoted the survival of B cells from approximately 40% of conventional Breg-inducing systems to nearly 80% (Figures S2C and S2D). Collectively, IL-10-producing Bregs with lower TNF-α expression could be vigorously induced by MSCs combined with GSK-3β inhibitor *in vitro*.

### The induced Bregs could be identified by a specific CD1c^+^ phenotype

**Figure 2:**
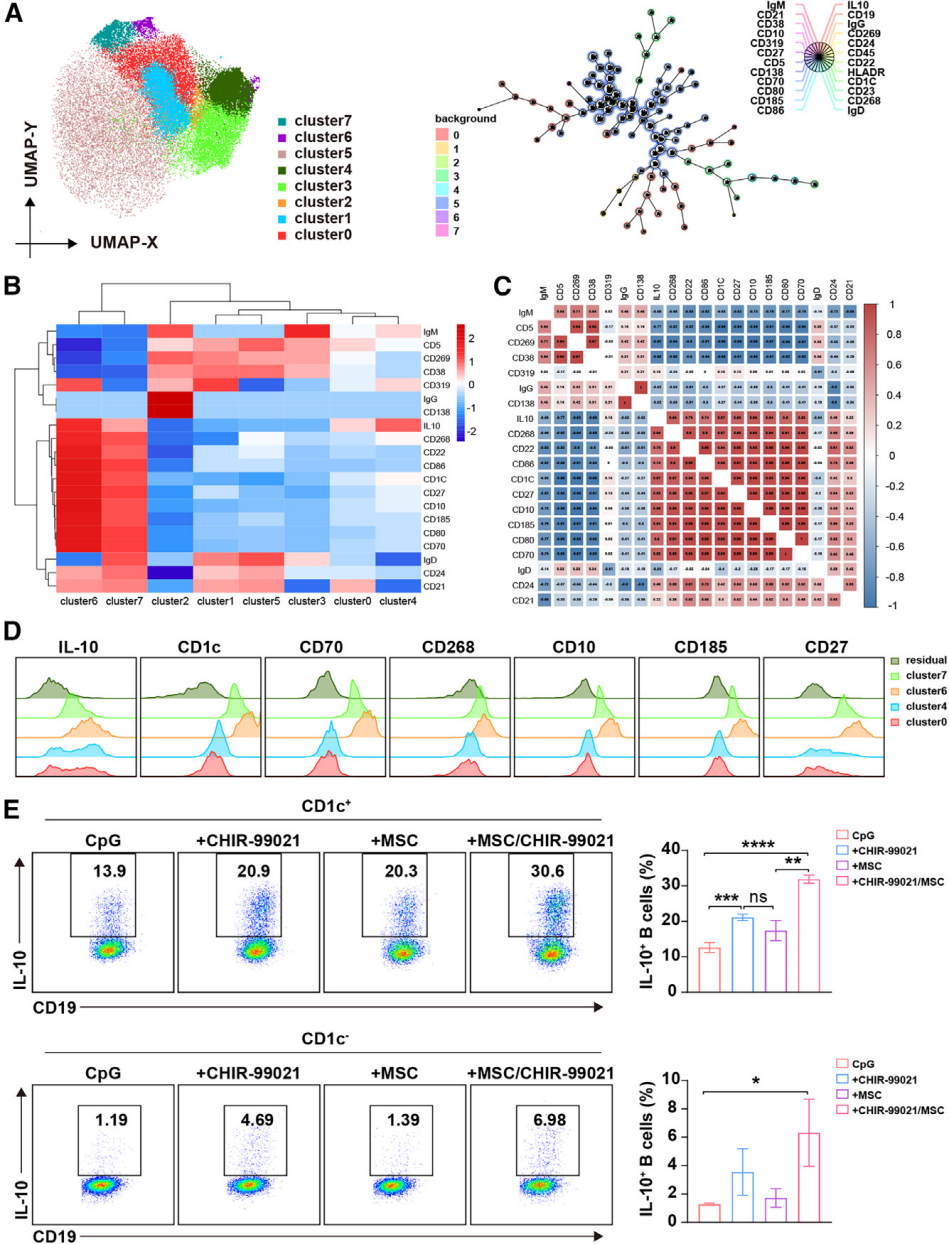
MSCs combined with CHIR-99021-induced IL-10^+^ B cells highly express CD1c B cells were cultured with MSCs and CHIR-99021 in the presence of CpG for 48 h. (A) Position projection of FlowSOM clusters in uniform manifold approximation and projection (UMAP) and the self-organizing map resulting from FlowSOM. (B) The mean fluorescence intensity (MFI) heatmap of key markers of B cells in FlowSOM clusters. (C) Pearson’s correlation coefficients of pairwise comparisons were calculated for key markers of B cells in FlowSOM clusters. (D) The histograms of IL-10, CD1c, CD10, CD27, CD70, CD185, and CD268 between IL-10^+^ B cell groups (cluster0, cluster4, cluster6, cluster7) and IL-10^−^ B cells (residual). (E) B cells were stimulated by CpG, MSCs, and CHIR-99021 individually or in combination for 48 h, and the frequency of CD1c^+^IL-10^+^ and CD1c^−^IL-10^+^ B cells was assessed by flow cytometry. Data represent mean ± SEM of three independent experiments; not significant (ns) *p* ≥ 0.05; ∗*p* < 0.05; ∗∗*p* < 0.01; ∗∗∗*p* < 0.001; ∗∗∗∗*p* < 0.0001.

It is well known that IL-10 is an intracellular cytokine and is not suitable for the enrichment or purification of Bregs for cell therapy. Thus, we further investigated the phenotypic characteristics of the Bregs that induced by MSCs combined with GSK-3β inhibitor. Using spectral flow cytometry, we explored the phenotype of these IL-10^+^ B cells via a 24-color B cell panel, including IL-10 and IL-23 surface markers for classification, activation, differentiation, cell state, and more. For unbiased analysis, B cells were clustered via the uniform manifold approximation and projection (UMAP) project, and the induced B cells were separated into different subsets (Figure S3A). Eight clusters of B cells were obtained by FlowSOM analysis, namely cluster0–cluster7, and IL-10^+^ B cells were mainly distributed in cluster0, cluster4, cluster6, and cluster7 (Figures 2A and 2B). To better explore suitable markers for the enrichment of IL-10^+^ B cells, we conducted the correlation analysis on the expression levels of major markers in cluster0–cluster7. The results showed that IL-10 had the strongest correlation with CD1c, reaching 0.87 (Figure 2C). Comparing the four clusters of IL-10^+^ B cells with residual IL-10^−^ B cells, we also found that CD1c had a similar expression pattern with IL-10, and CD1c showed better for distinguishing IL-10^+^ B cells from IL-10^−^ B cells (Figure 2D). These suggested that CD1c may be a general marker of IL-10^+^ B cells.

To further verify the relationship between CD1c and IL-10, we detected the expression patterns of CD1c and IL-10 by conventional flow cytometry and found that nearly all IL-10^+^ B cells are highly enriched in CD1c^+^ B cells (Figures S3B and S3C). Moreover, CD1c^+^ B cells significantly expressed more IL-10 than CD1c^−^ B cells, and CD1c^+^ was suitable for enriching the IL-10-producing B cells (Figure 2E). Since the IL-10:TNF-α ratio is usually used as a meaningful metric for evaluating the immunoregulatory capacity of functional Breg cells,^17^^,^^24^ we further quantified the log2 ratio of IL-10^+^:TNF-α^+^ and found that the B cells from our Breg induction system possessed the highest IL-10:TNF-α ratio. Importantly, the ratio of induced CD1c^+^ B cells is dramatically higher than that of the CD1c^−^ B cells (Figure S4), further indicating that CD1c is a good marker to enrich these IL-10 Bregs. In addition, the expression of CD1c is relatively stable during the induction process, and MSCs did not alter the expression of CD1c in B cells (Figure S3D), which suggests that CD1c expression was not induced. The induced Bregs could be identified by CD1c^+^ phenotype, and CD1c might be a marker for the B cell subsets, with the potential to secrete IL-10.

### MSC-mediated enhancement of IL-10 expression in B cells is dependent on PKA-CREB signaling

**Figure 3:**
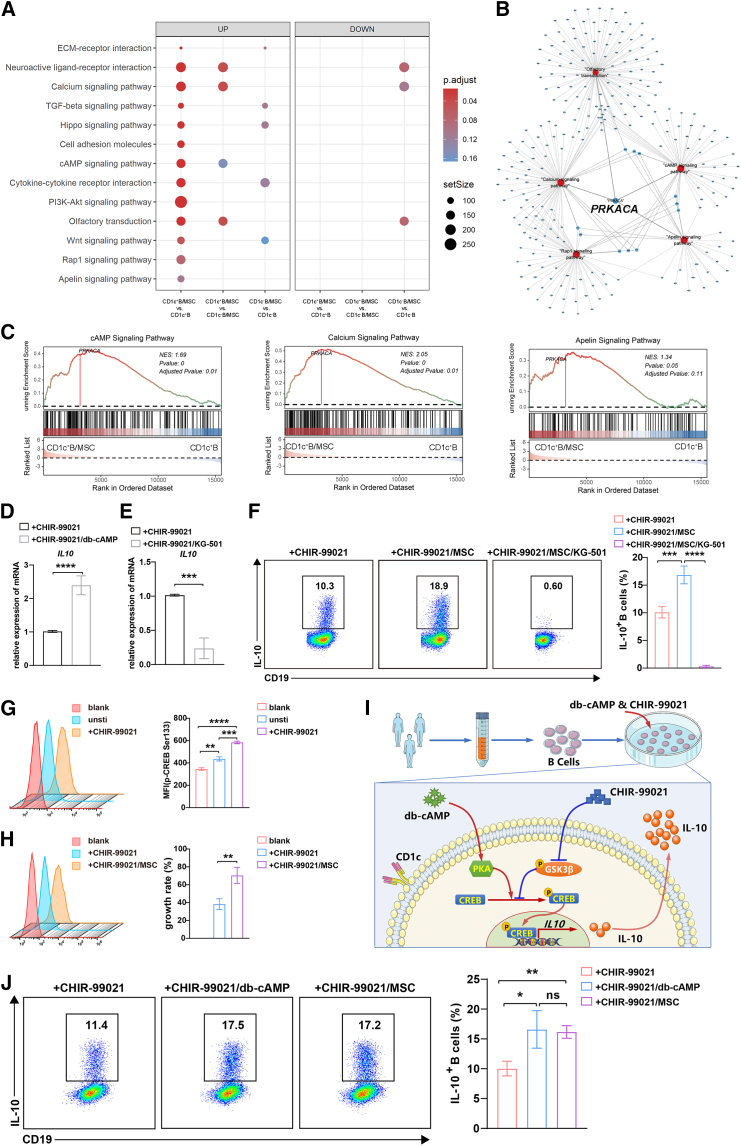
MSCs and CHIR-99021 induced IL-10^+^ B cells via PKA-CREB signaling of B cells (A) GSEA enrichment analysis of upregulated and downregulated signaling pathways. (B) The signaling pathways with their gene linkages. (C) Enrichment plots showed the three signaling pathways in CD1c^+^ B cells with MSCs compared with CD1c^+^ B cells individually. (D) Quantitative reverse-transcription-PCR (qRT-PCR) detection of the gene expression of *IL10* in B cells alone or with db-cAMP (a PKA agonist, 100 μM) in the presence of CpG and CHIR-99021. (E) qRT-PCR detection of the gene expression of *IL10* in B cells alone or with KG-501 (a CREB inhibitor, 10 μM) in the presence of CpG and CHIR-99021. (F) In the presence CpG and CHIR-99021, IL-10 production by B cells alone, B cells cocultured with MSCs, and B cells cocultured with MSCs and KG-501 were detected by flow cytometry. Quantification of IL-10-producing B cells. (G) Phosphorylation of CREB (Ser 133) in B cells alone or stimulated by CpG and CHIR-99021 was analyzed by flow cytometry, and MFI was used for statistical analysis. (H) Phosphorylation of CREB (Ser 133) in B cells alone or with MSCs in the presence of CpG and CHIR-99021 was analyzed by flow cytometry, and MFI was used for statistical analysis. (I) Optimization for a human Breg-inducing system based on PKA-CREB agonist and GSK3β inhibitor. (J) In the presence of CpG and CHIR-99021, IL-10 production by B cells alone, B cells cocultured with MSCs, and B cells treated with db-cAMP were detected by flow cytometry. Quantification of IL-10-producing B cells. Data represent mean ± SEM of three or more independent experiments. not significant (ns) *p* ≥ 0.05; ∗*p* < 0.05; ∗∗*p* < 0.01; ∗∗∗*p* < 0.001; ∗∗∗∗*p* < 0.0001.

To overcome the functional heterogeneity of MSCs in inducing Bregs, we further focused on the mechanism of IL-10 expression in B cells by MSCs. We sought to uncover the relevant pathways by which MSCs regulated CD1c^+^ B cells. Using GSEA analysis, we found that the cAMP signaling pathway, calcium signaling pathway, Apelin signaling pathway, and others were the upregulated pathway during MSC induction of CD1c^+^ B cells, while they were downregulated or absent during CD1c^−^ B cells induction by MSCs (Figure 3A). Interestingly, the crosstalk analysis of these signaling pathways showed that *PRKACA*, encoding the catalytic subunit of cAMP-dependent PKA, was the only shared gene (Figures 3B and 3C), suggesting that PKA might be a key regulator for inducing IL-10^+^ Bregs. Thus, dibutyryl cAMP (db-cAMP), a PKA agonist, was added to the Breg cell induction system without MSCs. db-cAMP significantly enhanced *IL10* mRNA expression (Figure 3D), supporting the role of PKA in inducing IL-10^+^ Bregs.

Next, we investigated how PKA regulated the IL-10 expression. As shown in Figure S5, we found that several transcription factors, which have been reported to regulate IL-10 expression, were involved in the PKA signaling by which MSCs regulated CD1c^+^ B cells. Among them, the cAMP-responsive element-binding protein (CREB) drew our attention. Many previous studies reported that CREB played a key role in balancing the inflammatory response, which was consistent with our results of inducing IL-10 while reducing TNF-α (Figures 1E, 3D, 3E, S2A, S2B, and S4). To elucidate whether PKA-CREB was involved in Breg induction, we blocked CREB by naphthol AS-E phosphate (KG-501), an inhibitor that disturbed CREB-dependent transcription. Interestingly, the IL-10 expression of B cells was remarkedly downregulated by KG-501 in the inducing systems without MSCs. Furthermore, the IL-10^+^ B cells nearly vanished in the MSC-based induction system (Figures 3E and 3F). Using a phospho-flow cytometry assay, we further confirmed that CREB of B cells was activated during this process, CHIR-99021 upregulated CREB phosphorylation at Ser133 in B cells (Figure 3G), and MSCs could further increase its phosphorylation (Figure 3H). Thus, CHIR-99021 and MSCs, in the MSC-based Breg-inducing system, synergistically promoted the activation of CREB in B cells, which contributed to upregulating the expression of IL-10 in CD1c^+^ B cells.

Based on the above findings, we tried to optimize the human Breg-inducing systems using the PKA/CREB agonist instead of MSCs (Figure 3I). As expected, db-cAMP combined with CHIR99021 can also effectively induce IL-10 expression and increase the percentage of IL-10^+^ B cells notably, with an induction efficiency that has reached a level comparable to that of MSCs (Figure 3J). db-cAMP not only induced Bregs but it also suppressed the TNF-α production of B cells in a dose-dependent manner (Figure S6). Collectively, IL-10-producing Bregs with lower TNF-α expression could be vigorously generated by a chemically defined inducing system *in vitro*.

### *Ex vivo*-yielded CD1c^+^ Bregs possessed the immunoregulatory capacity *in vitro*

**Figure 4:**
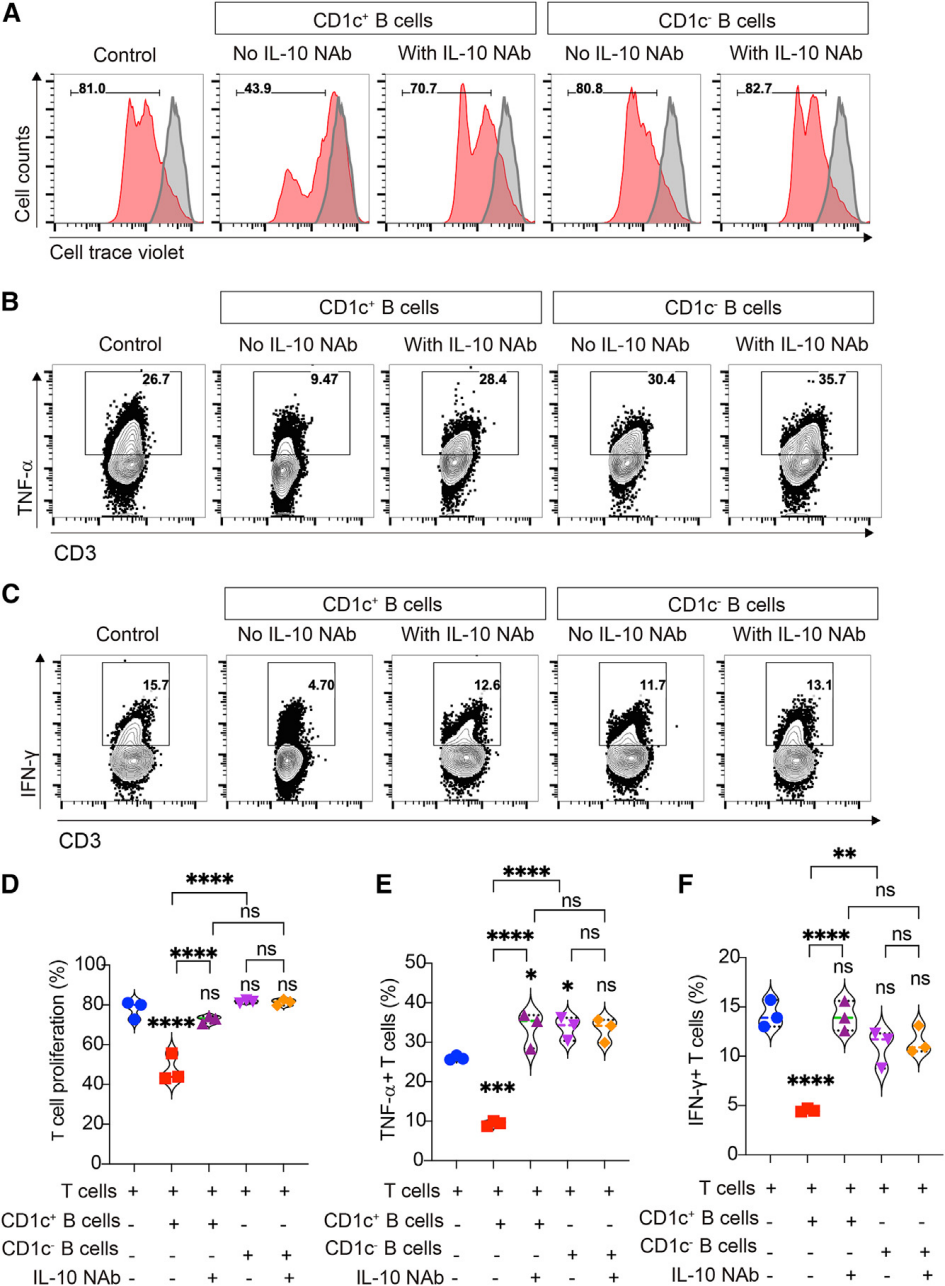
Induced CD1c^+^ B cells inhibited the proliferation of PBMCs and pro-inflammatory cytokine secretion of T cells IL-10-producing B cells were induced by db-cAMP and CHIR-99021 in the presence of CpG. Then, CD19^+^CD1c^+^ B cells and CD19^+^CD1c^−^ B cells were sorted and cocultured with PBMCs or CD3^+^ T cells. (A) Representative histograms of CellTrace Violet-labeled PBMC proliferation with or without B cells in the presence or absence of anti-IL-10 neutralizing antibody (IL-10 NAb), and the gray histogram is the unstimulated PBMC as control. (B) Representative plots of TNF-α production by T cells in the presence or absence of IL-10 NAb. (C) Representative plots of IFN-γ production by T cells in the presence or absence of IL-10 NAb. (D–F) The quantification of CellTrace Violet dilution (D), TNF-α-producing T cells (E), and IFN-γ-producing T cells (F). Data represent mean ± SEM of three independent experiments; not significant (ns) *p* ≥ 0.05; ∗*p* < 0.05; ∗∗*p* < 0.01; ∗∗∗*p* < 0.001; ∗∗∗∗*p* < 0.0001.

To check whether CD1c^+^ B cells yielded from our inducing system had immunoregulatory function, we isolated CD1c^+^ and CD1c^−^ B cells after induction and separately cocultured with CellTrace Violet (CTV)-labeled peripheral blood mononuclear cells (PBMCs). As shown in Figure 4A, PBMCs proliferated well after activation, while their proliferation was obviously inhibited when cocultured with CD1c^+^ B cells, but not CD1c^−^ B cells. Moreover, the inhibiting effects of CD1c^+^ B cells were reversed in the presence of anti-IL-10 neutralizing antibody (IL-10 NAb), indicating that induced CD1c^+^ B cells possessed the immunoregulatory capacity, which was mainly dependent on their expression of IL-10. Since the cytotoxic effects of T cells play an important role in the occurrence and development of GVHD, we further evaluated the effects of the induced CD1c^+^ Bregs on regulating the pro-inflammatory factors of T cells. The results showed that CD1c^+^ B cells were able to suppress the secretion of TNF-α and interferon-γ (IFN-γ) by CD3^+^ T cells, and IL-10 NAb could largely reverse their inhibitory effects. However, the suppressing effects of CD1c^−^ B cells were very weak and unstable (Figures 4B–4F). These observations indicated that the CD1c^+^ B cells yielded from our PKA/CREB axis-dependent inducing system possessed immunosuppressive functions, and these CD1c^+^ Bregs might be suitable for GVHD therapy.

### *Ex vivo*-yielded CD1c^+^ Bregs alleviated GVHD in a humanized mouse model

**Figure 5:**
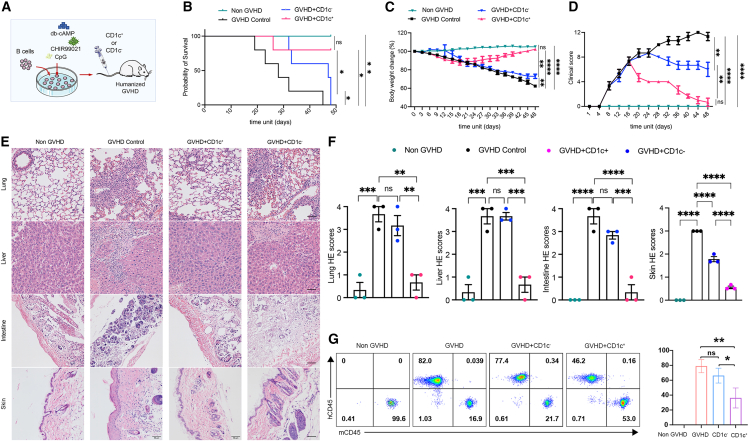
Adoptive transfer of induced CD1c^+^ B cells ameliorated humanized GVHD mice Humanized mice were injected with 1.5 × 10^7^ human PBMCs, followed by the administration of 1 × 10^6^ CD19^+^CD1c^+^ B cells (GVHD + CD1c^+^), CD19+CD1c^−^ B cells (GVHD + CD1c^−^), or vehicle administration (GVHD + PBS, as control). PBS was injected in the sham group instead of PBMCs (non-GVHD). (A) Schematic illustration of modeling and treatment of humanized GVHD mice model. (B–D) Comparison of survival rate, body weight loss, and clinical scores. (E and F) Histological examination of target organs, including skin, lung, liver, and intestine with H&E staining (scale bar, 50 μm) (E), and quantification of histological disease scoring in indicated groups (F). (G) Inhibition of T cell proliferation in GVHD mice infused with human CD1c^+^ B cells and CD1c^−^ B cells. Representative plots show the percentage of human (h) and mouse (m) CD45^+^ cells in the peripheral blood of GVHD mice in indicated groups, and quantification of the percentage of hCD45^+^ cells. Data represent mean ± SEM of three or more independent experiments; not significant (ns) *p* ≥ 0.05; ∗*p* < 0.05; ∗∗*p* < 0.01; ∗∗∗*p* < 0.001; ∗∗∗∗*p* < 0.0001.

To further investigate the GVHD therapeutic potential of the *ex vivo*-yielded CD1c^+^ Bregs, we next assessed their effects in treating GVHD in a humanized mouse model transplanted with human PBMCs. After confirming the successful implantation of PBMCs, the mice with GVHD were divided into three groups (Figure S7), respectively injected with induced CD1c^+^ B cells, CD1c^−^ B cells, or PBS (control) via tail vein (Figure 5A). Expectedly, all mice from the control group (GVHD) died within 50 days, while the GVHD mice that received CD1c^+^ B cells survived well, and the survival rate of mice that received CD1c^+^ B cells was remarkably higher than those that received CD1c^−^ B cells (Figure 5B). Furthermore, the data of weight loss (Figure 5C), GVHD clinical score (Figure 5D), and histopathologic damages (Figures 5E and 5F) confirmed that CD1c^+^ B cells generated from the PKA/CREB axis-dependent induction system can also alleviate the clinical symptoms, decrease inflammatory cell infiltration, and reduce tissue injury in mice with GVHD. However, injection of CD1c^−^ B cells showed poor improvement compared to CD1c^+^ B cells (Figures 5C–5F). In addition, flow cytometric analysis of PB demonstrated that *ex vivo*-yielded CD1c^+^ B cells decreased the percentage of human CD45^+^ cells (mainly T cells) in GVHD mice (Figure 5G), and obviously decreased the infiltration of T cells in the target organs as shown in immunohistochemical staining (Figure S8). However, poor improvement was observed in mice treated with CD1c^−^ B cells (Figures 5G and S8). Collectively, the PKA/CREB axis-dependent induction system was a feasible alternative Breg induction system that yielded a large number of CD1c^+^ Bregs with GVHD therapeutic potential.

## Discussion

A large number of studies have reported that B cells of different developmental stages,^25^ such as immature B cells, mature B cells, and plasmablasts, are able to differentiate into IL-10^+^ Bregs. IL-10^+^ Bregs are defined mainly by secreting IL-10, without specific makers or transcription factors like Tregs. Until now, multiple phenotypes, such as CD24^hi^CD27^+^, CD24^hi^CD38^hi^, and CD27^int^CD38^+^, have been reported to characterize the IL-10^+^ Bregs,^26^ further suggesting their diverse states. In our *ex vivo* induction system, we identified that IL-10^+^ B cells expressed high-level CD1c, which was suitable for the enrichment of the IL-10-producing B cells after induction. CD1c is a transmembrane glycoprotein, a member of the CD1 family. CD1c is not expressed in mice, but it is expressed in human normal, activated, and malignant B cells.^27^^,^^28^ CD1c was reported to be expressed on TIGIT^+^ (T cell immunoreceptor with immunoglobulin [Ig] and immunoreceptor tyrosine-based inhibitory motif domains-positive) human memory B cells, characterized as CD19^+^CD24^hi^CD27^+^CD39^hi^IgD^−^IgM^+^CD1c^+^.^29^ These TIGIT^+^ memory B cells express inhibitory molecules (including IL-10 and transforming growth factor β1) and inhibit T cell responses. Here, we found that CD1c might also serve as a marker for IL-10-producing Bregs and that the induced CD1c^+^ B cells possessed approximately 30% of IL-10^+^ cells, which is higher than those of other reported classical human Breg subsets, such as CD24^hi^CD27^+^ Bregs and CD24^hi^CD38^hi^ Bregs.^30^^,^^31^ The *ex vivo*-induced CD1c^+^ B cells possessed the immunoregulatory capacity *in vitro*, and ameliorated GVHD in a humanized mouse model. In addition, MSCs did not change the expression of CD1c, and CD1c^+^ B cells from PBMCs showed very high efficiency in the induction of Bregs. However, CD1c^−^ B cells were poorly induced (data not shown), suggesting that B cell subsets had the potential to secrete IL-10, mainly within the CD1c^+^ B cell subset. Whether CD1c is a functional molecule for IL-10 expression in B cells or just a surface marker should be further investigated.

As mentioned above, IL-10^+^ Bregs are able to generated from B cells at different developmental stages, and the differentiation of Bregs is not associated with the expression of special factors at the specific development stage, but is mainly dependent on the environment of B cells.^26^ Thus, Bregs are inducible, and a variety of stimuli have been found to induce Bregs.^32^ IL-10-producing B cells can be induced by TLR agonists, B cell receptor signaling, CD40 ligation, and exogenous cytokines, individually and in combination.^29^^,^^33^^,^^34^^,^^35^^,^^36^^,^^37^ However, the induction of IL-10 expression in B cells was reported that was accompanied by a marked increase in pro-inflammatory cytokines such as TNF-α,^17^ which in turn exerted a pro-inflammatory effect.^16^ In addition, animal-derived ingredients in some inducing systems, such as using Chinese hamster ovary cells, also limit its clinical translation. Here, based on the TLR9 agonist system, we established a human IL-10^+^ Breg induction system by PKA-CREB agonist combined with GSK-3β inhibitor, which significantly boosted the IL-10-producing B cells. Moreover, TNF-α expression was obviously suppressed during induction. Thus, the IL-10:TNF-α ratio, usually used as a meaningful metric for evaluating the immunoregulatory capacity of functional Breg cells,^17^ significantly increased in our induced Bregs. As expected, the induced IL-10^+^ Bregs had the capacity to inhibit effector T cell proliferation and inflammatory cytokine release. Furthermore, adoptive transfer of these Bregs relieved the pathological symptoms of humanized GVHD mouse models. This has important implications for the development of Breg-based therapies for GVHD.

TLR agonists are well-defined signals to induce IL-10-producing B cells in both mice and humans.^38^ Upon TLR stimulation, several signaling pathways are activated, including nuclear factor κB (NF-κB), mitogen-activated protein kinase, and PI3K-Akt pathways. These pathways could converge on the activation of mitogen- and stress-activated protein kinase 1 (MSK1) and MSK2, which phosphorylate CREB, thereby driving IL-10 transcription.^39^ However, the activation of the TLR signaling pathway can also phosphorylate NF-κB component p65 to interact with CREB-binding protein (CBP)/p300 at the same region as phosphorylated CREB, thus not only promoting the release of pro-inflammatory cytokines such as TNF-α, IL-1β, IL-6, and IL-12 but also weakening the effect of CREB.^40^ In our *ex vivo* Bregs induction system, MSCs upregulating Wnt signaling or CHIR99021 could enhance IL-10 expression, potentially disrupting the inactivation of CREB by GSK-3β and the inhibition of CREB binding to CBP.^41^ Therefore, phosphorylated CREB competed with NF-κB for binding to CBP, leading to reduced pro-inflammation cytokine TNF-α production and enhanced anti-inflammatory cytokine IL-10 production. More important, the PKA-mediated phosphorylation of CREB on Ser 133 in CD1c^+^ B cells by MSCs or db-cAMP further enhanced the CREB activity to increase IL-10 transcription, while it decreased TNF-α.^42^ Collectively, the balance of CREB and p65 binding to CBP/p300 determines whether the induced B cells exhibit pro-inflammatory or anti-inflammatory effects, and the regulation of CREB activity by both PKA signaling and GSK3β inhibition could be an important strategy to obtain human IL-10^+^ B cells *ex vivo*.

### Conclusions

This study established an efficient induction system for IL-10^+^ Bregs based on MSCs combined with GSK-3β blockage, and found that CD1c^+^ B cells could enrich the induced Bregs. Moreover, to overcome the functional heterogeneity of MSCs, we successfully revealed that the PKA-mediated phosphorylation of CREB was the pivotal modulator for inducing Bregs in MSC-based systems. Thus, we developed a chemically defined inducing protocol for Bregs by PKA-CREB agonists, instead of MSCs, which can also effectively induce CD1c^+^ Bregs with lower TNF-α expression. Induced CD1c^+^ Bregs had immunosuppressive function and exhibited a powerful effect on alleviating GVHD. This study has created a scheme for the acquisition of human Bregs *in vitro* and provided a new idea for the prophylaxis and treatment of GVHD.

## Materials and methods

### Isolation and characterization of MSCs

MSCs were obtained from the bone marrow of healthy donors after obtaining informed consent. As previously described,^8^ marrow mononuclear cells were isolated by Ficoll-paque (Amersham Biosciences, Sweden) density gradient centrifugation and seeded at a density of 1 × 10^5^/cm^2^ into T75 cell culture flasks (CellBIND, Corning, USA). When cells grew to 80% confluence, they were detached by trypsin-EDTA designated as passage 1. These cells were further passaged at a ratio of 1:3. As shown in Figure S9, MSCs used in this study highly expressed the surface markers CD29, CD44, CD73, CD90, CD105, and CD166, but not CD34, CD45, and HLA-DR. Moreover, MSCs had a capacity to differentiate into osteoblasts, adipocytes, and chondroblasts. These characteristics were consistent with the criteria for the identification of MSCs.^43^^,^^44^ The well-characterized 5th–6th passage MSCs were used for the experiments.

### Cell sorting and culture

PBMCs of healthy donors were isolated by density gradient centrifugation using Ficoll (TBD, China). CD19^+^ B cells, CD19^+^CD1c^+^ B cells, CD19^+^CD1c^−^ B cells, and CD3^+^ T cells were purified by fluorescence-activated cell sorting. Cells were incubated in RPMI-1640 medium (GIBCO, USA) supplemented with 10% fetal bovine serum (FBS) (Hyclone, USA), 1% nonessential amino acids (GIBCO, USA), and 1% penicillin and streptomycin (GIBCO, USA).

Purified B cells were stimulated with 4 μg/mL CpG ODN 2006 (InvivoGen, USA) and 10 μM CHIR-99021 (an inhibitor of GSK-3β; Selleck, USA) in the presence or absence of MSCs. To explore the mode of action in this process, 1 μM KG-501 (an inhibitor of cAMP response element-binding protein, TargetMol, USA) was added to the B cell and MSCs coculture system, and 100 μM db-cAMP (a selective PKA activator; TargetMol, USA) was added to B cells cultured alone.

### Antibodies and flow cytometry

The human-specific antibodies used in this study included anti-human CD19 (phycoerythrin [PE]-Cy7, BV421, PE), anti-human CD20 (BV421), anti-human CD1c (BV421, BUV805), anti-human CD3 (fluorescein isothiocyanate [FITC], V450), anti-human CD4 (FITC), anti-human CD5 (PE-Cy5), anti-human CD27 (PE-Cy7), anti-human CD70 (BV786), anti-human CD138 (PE), anti-human CD269 (AF647), anti-human CD319 (PE/Dazzle 594), anti-human IgM (eFluor 450), anti-human IgD (BV510), anti-human IL-10 (PE-Cy7, eFluor 660, allophycocyanin [APC]), anti-human TNF-α (PE), anti-human IFN-γ (APC), and anti-human p-CREB (PE). Antibodies were purchased from BD Bioscience, eBioscience, BioLegend, and Invitrogen (all USA). Sufficient samples were analyzed using an Aurora (Cytek, USA) and a Cytoflex (Beckman Coulter, USA) flow cytometers. Data were analyzed using FlowJo, SpectroFlo, and CytExpert.

For intracellular cytokine staining, the Leukocyte Activation Cocktail (2 μL/mL, BD Biosciences, USA) was added in the last 5 h. Cells were washed and incubated with indicated antibodies for 20 min in the dark. Then, the cells were washed and fixed by 4% paraformaldehyde (Phygene, China) for 20 min. The cells were washed, permeabilized using 0.2% saponin (Sigma-Aldrich, USA), and incubated with anti-human IL-10-eFluor 660 or anti-human TNF-α-PE and anti-human IFN-γ-APC for 30 min in the dark. After washing, the samples were analyzed using a Cytoflex flow cytometer. Data were analyzed using FlowJo and CytExpert.

### Quantitative reverse-transcription-PCR analyses

According to the manufacturer’s protocol, total RNA was extracted from cell samples using a RNeasy Mini Kit (Qiagen, Germany). Then, cDNA was synthesized using the Reverse Transcription Kit (Novoprotein, China). We used *18S* as a control gene, and the gene expression of *IL10* was determined using SYBR qPCR Master Mix (Roche, Switzerland). Signals were tested using a Light Cycler 480 detection system (Roche). CT values were calculated in relation to 18S CT values by the $2^{-\Delta \Delta \text{CT}}$. All assays were repeated in three independent experiments. The primer sequences are described in Table S1.

### Suppression assay *in vitro*

For the proliferation assay, PBMCs were labeled with 5 μM CTV (CellTrace Violet Cell Proliferation Kit; Invitrogen, USA) and cocultured with CD19^+^CD1c^+^ or CD19^+^CD1c^−^ treated by CpG ODN 2006, db-cAMP, and CHIR-99021. PBMCs were stimulated with anti-CD3 monoclonal antibody (mAb) (1 μg/mL) and anti-CD28 mAb (1 μg/mL, BD Biosciences). PBMC proliferation was evaluated by flow cytometric analysis of CTV dilution.

### Humanized GVHD animal model and pathological examination

Highly immunodeficient B-NDG mice were purchased from Zhuhai BesTest Bio-Tech Company and were fed in a facility free of specific pathogens. All the mice were female and 6 to 8 weeks old. The animal experiment protocols were approved by the Institutional Animal Care and Use Committee of Sun Yat-sen University (SYSU-IACUC-2022-000338).

Human PBMCs (1.5 × 10^7^) were washed and transferred into mice via the tail vein on day 0. Then, tail vein blood was collected in heparin, and the proportion of human CD45 was detected to confirm the success of GVHD models on day 18. At day 21, 1 × 10^6^ CD19^+^CD1c^+^ or CD19^+^CD1c^−^ B cells treated by CpG ODN 2006, db-cAMP, and CHIR-99021 *in vitro* were injected into mice via the tail vein. PBS alone was used as a control. The weight of the mice was recorded every 3 days. After 50 days, major targeted organs (skin, lung, liver, and small intestine) were harvested in all groups, fixed in 4% paraformaldehyde, and paraffin embedded. The tissue sections were stained with H&E. PB was collected for donor T cell proliferation using flow cytometry, as previously described.^45^

### Assessment of mice GVHD

The severity of mice GVHD was assessed according to a clinical scoring system for mice with GVHD, including six clinical criteria: weight loss, diarrhea, posture, activity, fur texture, and skin integrity. Each item was scored as 0–2 according to the manifestation. The total GVHD score of each mouse was measured in a 4-day interval. Details of the clinical scoring system for mice with GVHD is shown in Table S2. Histological scoring of GVHD target tissues was performed according to the level of lymphocyte infiltration. The lymphocyte infiltration score was determined as follows: 0, normal; 0.5, focal and rare; 1, focal and mild; 2, diffuse and mild; 3, diffuse and moderate; 4, diffuse and severe.

### Phosphoflow assay

According to the manufacturer’s protocol, all B cells were harvested and washed with 2% FBS/PBS. Then, the cells were lysed and fixed immediately with 1× BD Phosflow Lyse/Fix Buffer (BD Pharmingen, USA) at 37°C for 15 min. Centrifuged at 600 × *g* for 5 min, we removed the supernatant and vortexed it to disrupt the cell pellet. The cells were permeabilized with BD Phosflow Perm Buffer II (BD Pharmingen) on ice for 30 min. Then, they were washed twice and stained with anti-CREB-PE at room temperature for 60 min in the dark. After washing, the samples were analyzed using a Cytoflex flow cytometer (Beckman Coulter). Data were analyzed using FlowJo.

### Processing and analysis of RNA-seq data

Raw fastq files were processed and analyzed following the methodology described herein. Briefly, raw fastq files were aligned and quantified against the GRCh38 reference genome using default parameters in STAR^46^ (version 2.7.11b) and RSEM^47^ (version 1.3.3), respectively, to generate counts. All subsequent analyses were conducted in the R computing environment.

Differential expression analysis was conducted using the default workflow of DESeq2^48^^,^^49^ (version 1.42.0) and edgeR^50^ (version 4.0.16). To interpret the genome-wide expression profiles, GSEA was applied as a knowledge-based approach. Genes were ranked based on their expression values, and enrichment analysis was performed using either the C2 gene set from MSigDB^32^ (version 7.5.1) or Kyoto Encyclopedia of Genes and Genomes gene sets.^51^ The R package clusterProfiler (version 4.10.0)^52^ and ggplot2 (version 3.5.1) were utilized for enrichment analysis and result visualization. Visualization of the GSEA results was achieved using the GseaVis package.

### Statistical analysis

Data were statistically analyzed using the two-tailed Student’s t test and one-way ANOVA. All analyses were performed using GraphPad Prism 9.0 software (GraphPad, USA). *p* < 0.05 was set as the statistical significance.

## Data and code availability

All data associated with this study are present in the paper or in the supplemental information. Sequencing data have been deposited at GEO and is accessible under GEO: GSE279879. Additional data related to this paper may be requested from the authors.

## Acknowledgments

This work was supported by grants from the 10.13039/501100012166National Key Research and Development Program of China, 10.13039/501100013290Stem Cell and Translational Research (2022YFA1105000, 2022YFA1104100); the 10.13039/501100001809National Natural Science Foundation of China (82270230, 81970109, 32130046); the 10.13039/501100021171Guangdong Basic and Applied Basic Research Foundation (2023B1515020119); the Key Scientific and Technological Program of Guangzhou City (2023B01J1002); the Pioneering Talents Project of Guangzhou Development Zone (2021-L029); the Shenzhen Science and Technology Program (KJZD20230923114504008); the Sanming Project of Medicine in Shenzhen Nanshan (no. SZSM202103012); and the Shenzhen Nanshan District Science and Technology Program (NS2022023, NS2023012, NSZD2024048, NSZD2024051), the Science and Technology Planning Project of Gaozhou (20240619111622). The graphical abstract and some elements in Figures 1, 3, and 5 were generated using Servier Medical Art (https://smart.servier.com/), licensed under a Creative Commons Attribution 4.0 Unported License.

## Author contributions

Y.B., J.L., Z.L., and Y.S. designed the experiments, performed the research, and wrote the manuscript. J.C., Z.L., and X.G. collected the animal samples. Y.S. and Y.M. contributed to the RNA-seq data analysis. G.L., T.W., H.L., X.Z., R.Y., R.F., and J.F. performed the samples collection and cell isolation. X.C., A.P.X., and W.X. designed and supervised the study, interpreted the data, and wrote the manuscript. All authors approved the manuscript and give their consent for submission and publication.

## Declaration of interests

The authors declare no competing interests.

## Supplemental information

Document S1. Figures S1–S9, Tables S1, and S2

Document S2. Article plus supplemental information
