# Supplementary material for: Ex vivo-generated human CD1c+ regulatory B cells by a chemically defined system suppress immune responses and alleviate graft-versus-host disease
Source: Mol Ther. 2024 Oct 26;32(12):4372–82. doi: 10.1016/j.ymthe.2024.10.026 (PMC11638867; doi:10.1016/j.ymthe.2024.10.026)
Supplement: Document S1. Figures S1–S9, Tables S1, and S2 [file mmc1.pdf]

YMTHE, Volume *32*

Supplemental Information

Ex vivo-generated human CD1c$^+$ regulatory B cells by a chemically defined system suppress immune responses and alleviate graft-versus-host disease

Yingying Bao, Jialing Liu, Zhishan Li, Yueming Sun, Junhua Chen, Yuanchen Ma, Gang Li, Tao Wang, Huanyi Liu, Xiaoran Zhang, Rong Yan, Zhenxia Yao, Xiaolu Guo, Rui Fang, Jianqi Feng, Wenjie Xia, Andy Peng Xiang, and Xiaoyong Chen

Supplemental Materials

Supplemental Figures

Figure S1

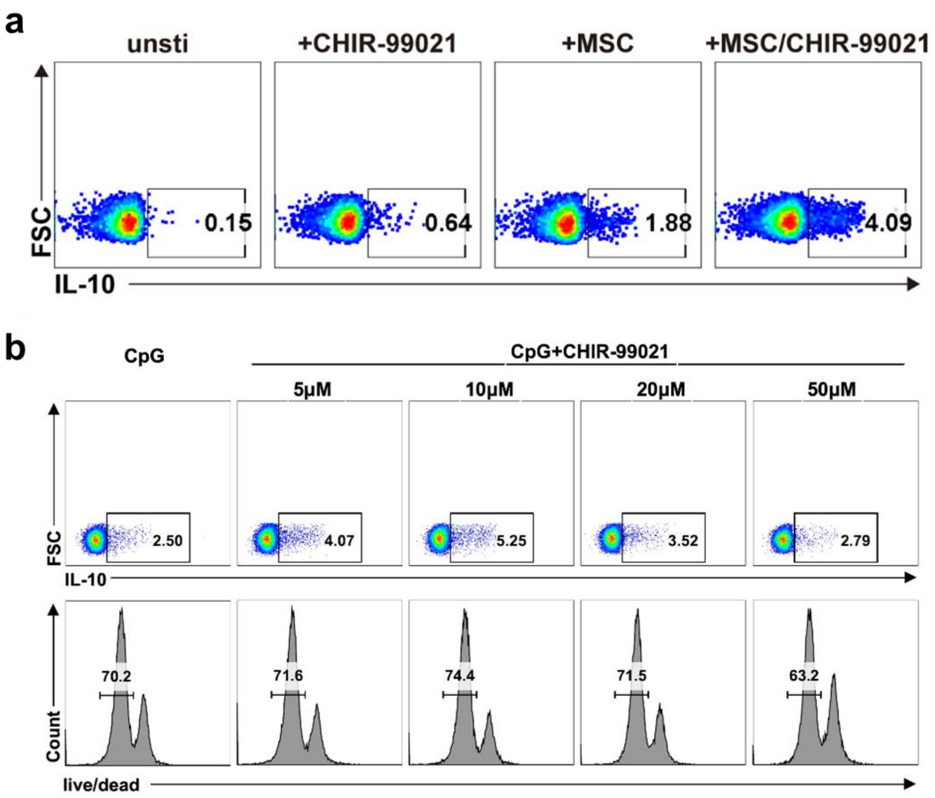

Figure S1. CHIR-99021 have capacity to induce IL-10^{+} B cells. (a) IL-10 production by B cells, stimulated by MSCs and CHIR-99021 individually or in combination, was detected by flow cytometry. (b) IL-10 production and the survival of B cells in the CpG system with different concentrations of CHIR-99021 were detected by flow cytometry.

Figure S2

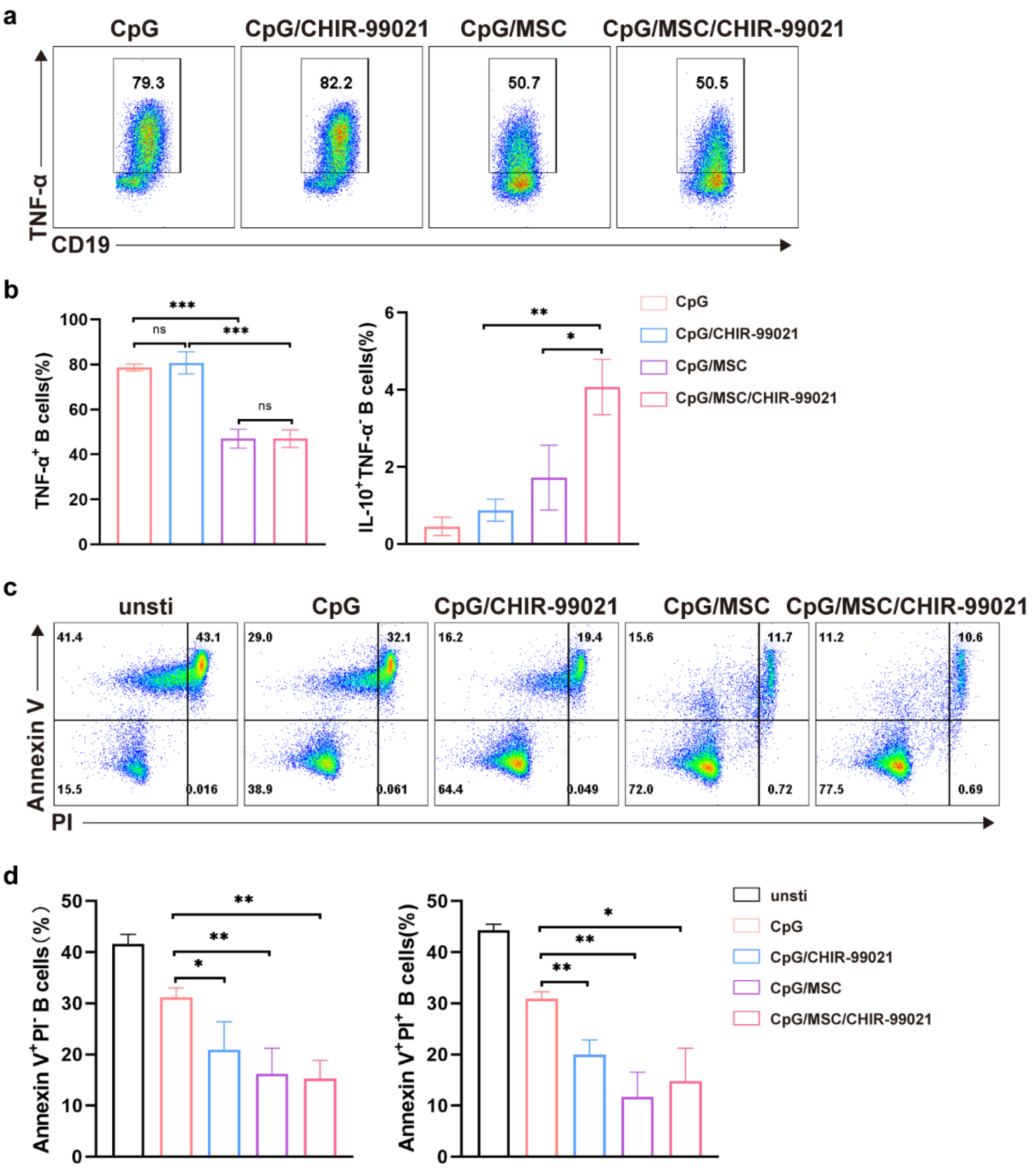

Figure S2. MSCs and CHIR-99021 inhibit TNF-α production of B cells and promote survival of B cells. (a) TNF-α production by B cells, stimulated by CpG, MSCs, CHIR-99021 individually or in combination, was detected by flow cytometry. (b) Quantification of TNF-α-producing B cells and the percentage of TNF-α$^-$IL-10$^+$ B cells in above treatment. (c) Apoptosis of B cells, stimulated by CpG, MSCs and CHIR-99021 individually or in combination, was detected by flow cytometry of Annexin

V/PI-stained. (d) Quantification of apoptosis (Annexin $V^{+}PI^{-}$) B cells and necrosis (Annexin $V^{+}PI^{+}$) B cells in above treatment. Data represent mean ± SEM of 3 independent experiment. not significant (ns) $p \geq 0.05$, *$p < 0.05$, **$p < 0.01$, ***$p < 0.001$, ****$p < 0.0001$.

Figure S3

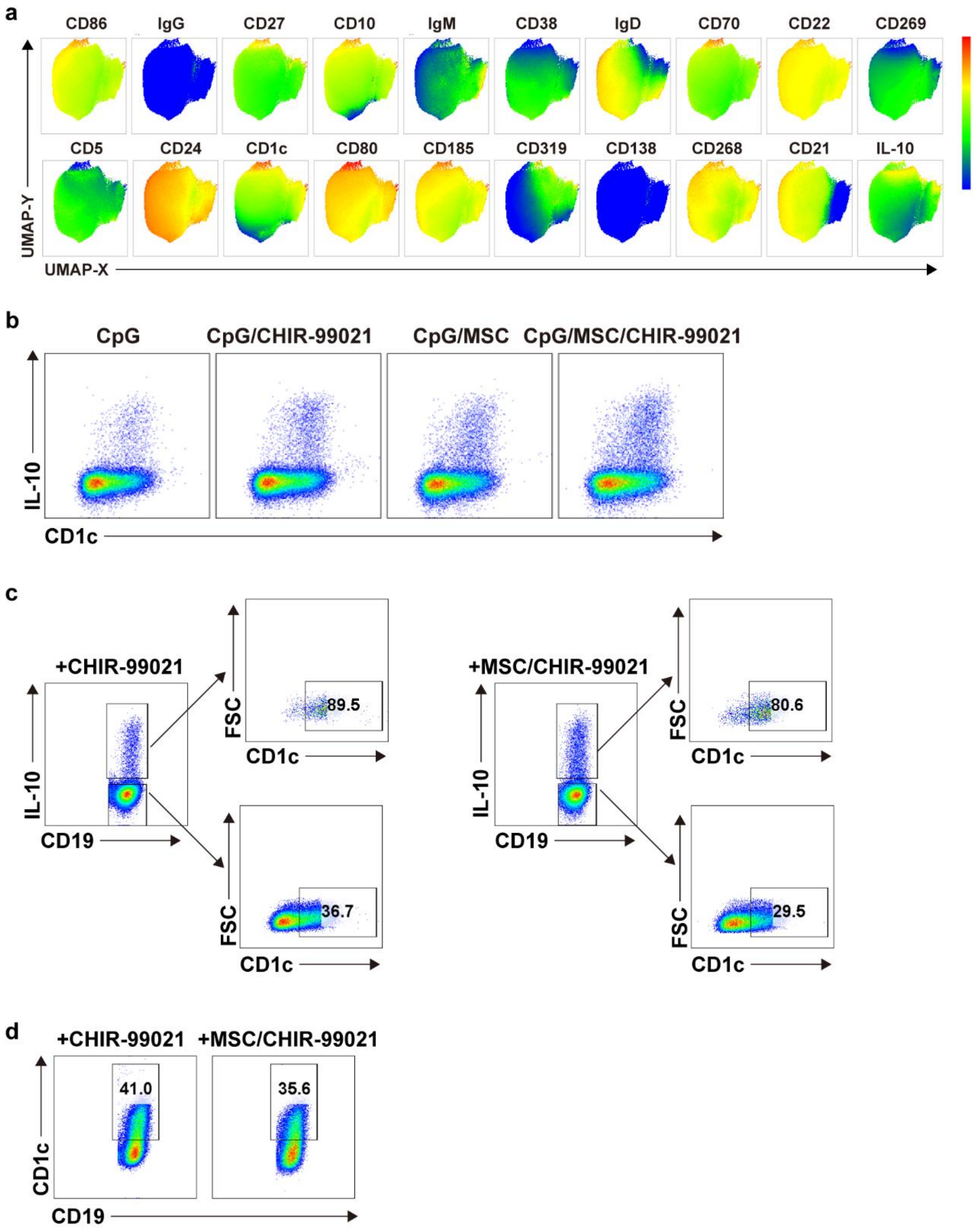

Figure S3. MSCs and CHIR-99021 induced IL-10^{+} B cells highly express CD1c. (a) UMAP clustering analysis of B cells co-culture with CpG, MSCs and CHIR-99021. (b) The expression of CD1c and IL-10 on B cells, stimulated by CpG, MSCs and CHIR-99021 individually or in combination, was detected by flow cytometry. (c) The

expression of CD1c on IL-10$^+$ and IL-10$^-$ B cells co-cultured with or without MSCs in the presence of CpG and CHIR-99021. (d) The expression of CD1c on CD19$^+$ B cells co-cultured with or without MSCs in the presence of CpG and CHIR-99021.

Figure S4

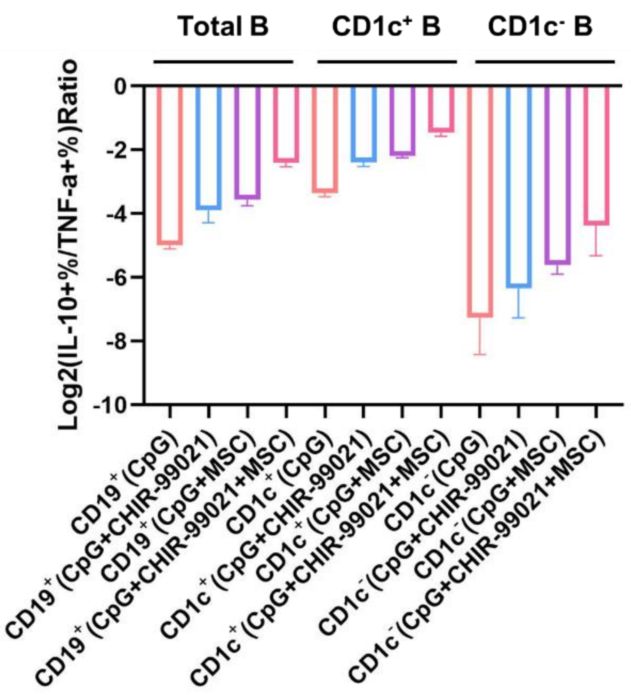

Figure S4. CD1c$^+$ B cells possessed a highest log2 ratio of IL-10$^+$/TNFa$^+$ of B cells. B cells stimulated by CpG, MSCs and CHIR-99021 individually or in combination, and the log2 ratio of IL-10$^+$/TNFa$^+$ of B cells in the inducing systems was calculated.

Figure S5

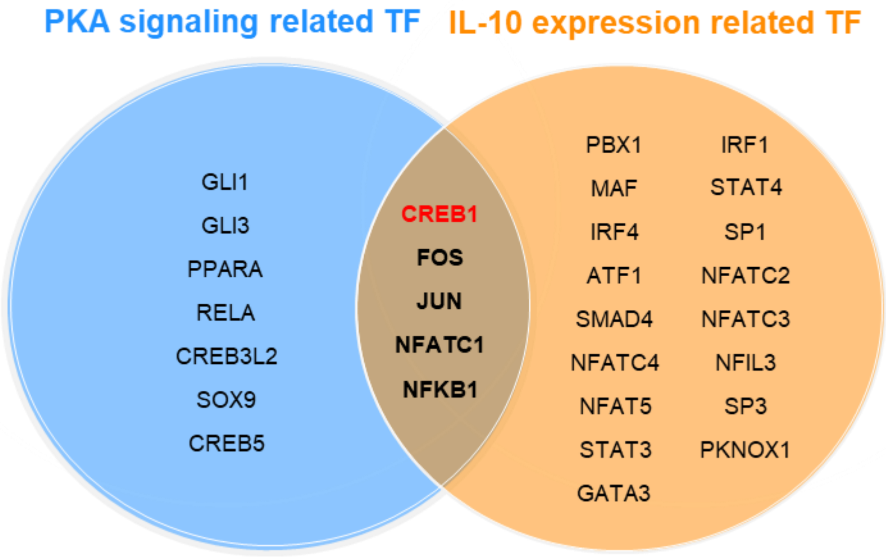

Figure S5. Screening for transcription factors involved in PKA signaling and regulating IL-10 expression. Venn diagram showed the transcription factors. Transcription factors that involved in PKA signaling of the MSC-induced $CD1c^{+}$ B cells was show in the blue pie chart, and the transcription factors reported to regulate IL-10 expression was shown in the orange pie chart.

Figure S6

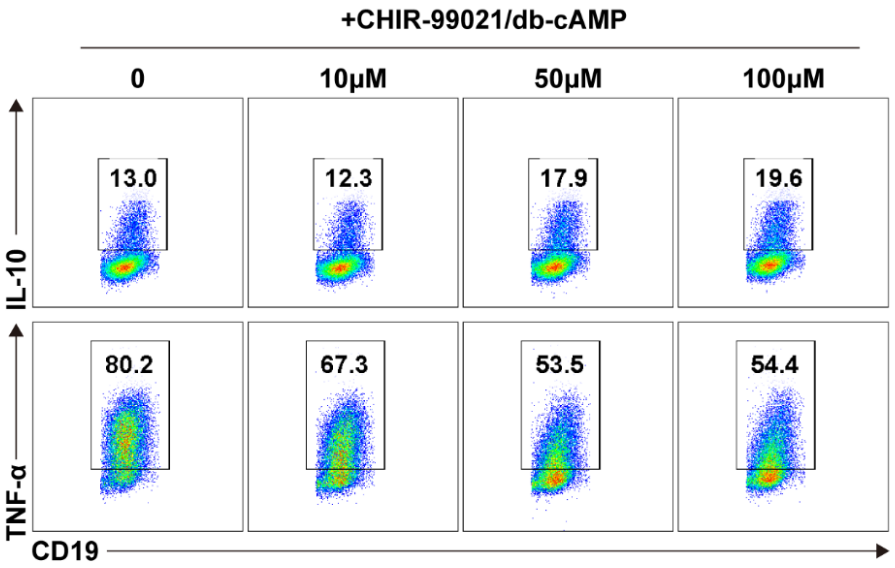

Figure S6. Activation of CREB inhibits TNF-α production and promotes IL-10 production by B cells. IL-10 and TNF-α production by B cells stimulated by different concentration of db-cAMP were detected by flow cytometry.

Figure S7

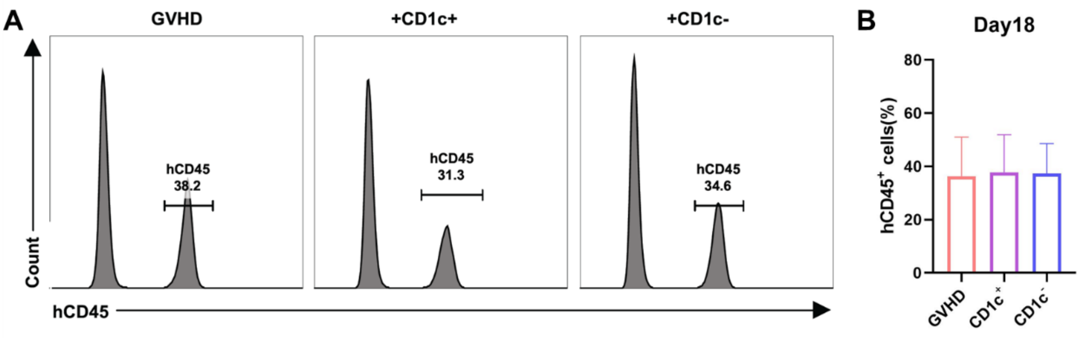

Figure S7. Successful engraftment of human PBMC in mice with GVHD.

Representative plots of hCD45^{+} cells of GVHD mice in different groups (A), and the quantification of hCD45^{+} cells (B).

Figure S8

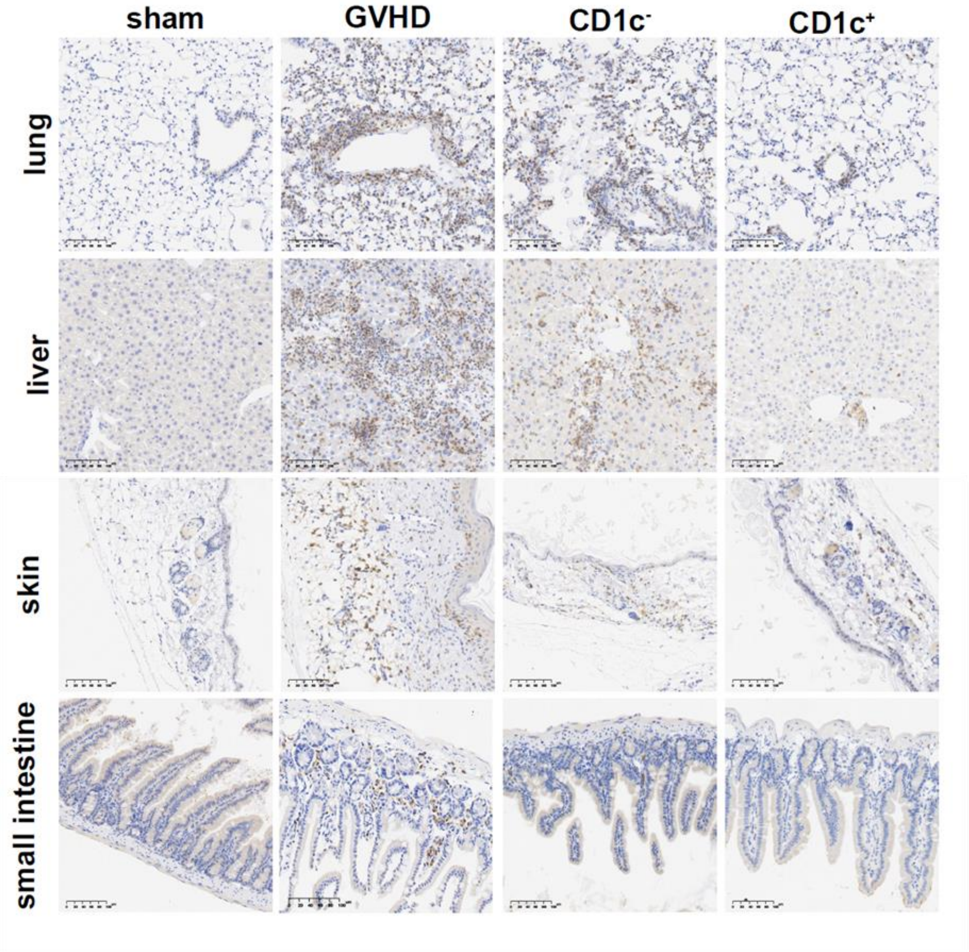

Figure S8. Induced CD1c+ B cells inhibit the infiltration of human CD3+ T cells.

The percentage of human CD3+ T cells was evaluated by immunohistochemical staining (scale bar, 100μm) in target organs of GVHD.

Figure S9

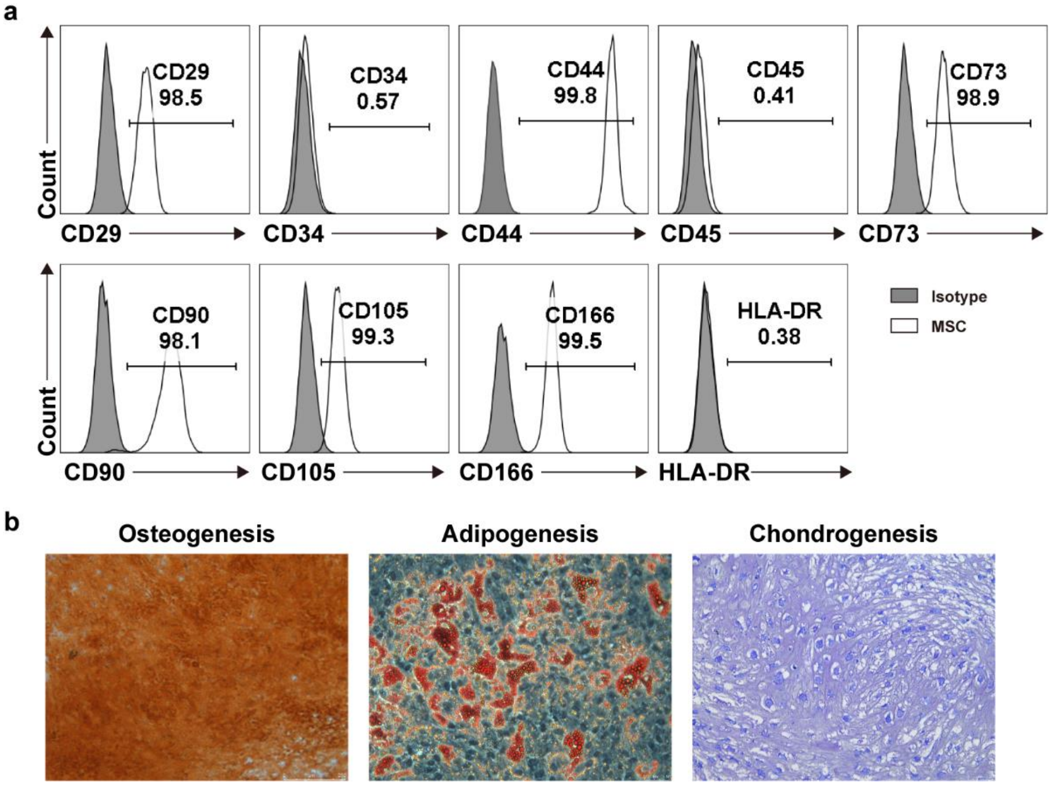

Figure S9. Identification of Human Bone morrow MSCs. (a) The expression of CD29, CD34, CD44, CD45, CD73, CD90, CD105, CD166 and HLA-DR on MSCs was detected by flow cytometry. (b) Oil red O, Alizarin red S, and Toluidine blue O staining were used to assess the osteogenesis, adipogenesis, and chondrogenesis of MSCs.

Supplemental Tables

Table S1. List of primer sequences

Names	Primers (5' to 3')
18S-F	CCCGAAGCGTTTACTTTGA
18S-R	CAAATGCTTTCGCTCTGGT
IL10-F	TCAAGGCGCATGTGAACTCC
IL10-R	GATGTCAAACTCACTCATGGCT

Table S2. Mouse GvHD Clinical Scoring System.

Grade	Weight Loss	Diarrhea	Posture	Activity	Fur Texture	Skin Integrity
0	<10%	No	Normal	Normal	Normal	Normal
1	>10% to <25%	Yes	Hunching at rest	Mild to moderate decrease	Mild to moderate rufflings	Scaling of paw and tails
2	>25%		Severely hunching	Severe decrease	Severe rufflings	Obviously denuded
